# Current State Analysis of Malnutrition Screening for Ambulatory Patients With Inflammatory Bowel Disease Reveals Low Screening Rates and Telehealth as a Risk Factor

**DOI:** 10.1016/j.gastha.2025.100870

**Published:** 2025-12-24

**Authors:** Bita Shahrvini, Andrew Chang, Alexandra C. Greb, Mark Baniqued, Divya P. Prajapati, Rhett Harmon, Sureya F. Hussani, Nirupama Bonthala, Gaurav Syal, Jenny S. Sauk, Folasade P. May, Berkeley N. Limketkai

**Affiliations:** 1 Vatche and Tamar Manoukian Division of Digestive Diseases, Department of Medicine, David Geffen School of Medicine at UCLA, Los Angeles, California; 2 Internal Medicine, Olive View University of California Los Angeles Medical Center, Los Angeles, California; 3 Division of Clinical Nutrition, Department of Medicine, David Geffen School of Medicine at UCLA, Los Angeles, California

**Keywords:** Crohn’s disease, Inflammatory bowel disease, malnutrition, telehealth, ulcerative colitis

## Abstract

**Background and Aims:**

Patients with inflammatory bowel disease (IBD) are at increased risk of malnutrition, which is associated with worse outcomes and has prompted recommendations for regular nutrition screening. This study details a current state analysis of outpatient gastroenterology (GI) malnutrition screening practices for patients with IBD and evaluates risk factors for lack of screening.

**Methods:**

This retrospective cohort study included adults with IBD on advanced therapies seen at the University of California, Los Angeles, between 2018 and 2024. Patient data were abstracted from outpatient GI encounters via electronic medical records. A root cause analysis for lack of malnutrition screening was created using a Gemba walk and stakeholder interviews. Multivariable logistic regression evaluated risk factors for lack of screening.

**Results:**

Of 283 included patients, the mean age was 44.4, mean body mass index was 25.9, 53.7% were female, 62.9% were White, and 50.0% had Crohn’s disease. Most (70.7%) had their GI encounters via telehealth. Malnutrition screening was performed at 56% of encounters. When patients were screened, a validated screening tool was used in 12% of encounters. Screening identified malnutrition risk in 11% of encounters and prompted ordering of registered dietician referrals 44% and nutrition labs 56% of the time. Malnutrition screening was less likely if the encounter was via telehealth (vs in-person, odds ratio 0.43, confidence interval [0.23–0.80]).

**Conclusion:**

Improved malnutrition screening among GI physicians for IBD patients is needed. Given telehealth visits were strongly associated with lack of screening, strategies to address this care gap are needed since telehealth has become more common.

## Introduction

Patients with inflammatory bowel disease (IBD) have an increased risk of malnutrition.^1^^,^^2^ The prevalence of malnutrition has been reported to be as high as 50% among ambulatory patients with IBD and it presents as protein–calorie malnutrition, micronutrient deficiencies, and altered body composition.^2^^,^^3^ Decreased oral intake, chronic inflammation, and malabsorption from both the disease process itself and treatments have all been proposed mechanisms for the increased rates of malnutrition in patients with IBD.^4^ Malnutrition in patients with IBD has been associated with poor clinical outcomes including increased rates of disease flares, hospitalizations, infections, postoperative complications, and mortality.^2^^,^^5^, ^6^, ^7^ Studies have shown that targeted nutritional interventions, such as enteral nutrition, parenteral nutrition, whole-food-based interventions, and targeted exclusion diets in patients with IBD that are malnourished can improve their clinical outcomes and help reduce inflammation.^8^^,^^9^ The European Society for Clinical Nutrition and Metabolism and American Gastroenterological Association thus recommends that all patients with IBD be screened for malnutrition risk both at time of diagnosis and regularly thereafter.^6^^,^^10^ Of note, while there are some American Gastroenterological Association guidelines for the United States that discuss importance of regular nutrition screening for patients with IBD, overall, there is lack of clarity on how to implement these general recommendations and with what frequency.

Although patients with IBD and providers alike cite nutrition as important to IBD management,^11^ standardization of malnutrition screening for ambulatory patients with IBD has not yet been widely adopted, in part because of lack of agreement on how to best conduct screening. Many existing malnutrition screening tools (MSTs) were designed to screen hospitalized patients.^11^^,^^12^ The Malnutrition Universal Screening Tool (MUST) and the MST are among the most commonly used instruments to screen for malnutrition in all populations.^13^ The Malnutrition Inflammation Risk Tool and Saskatchewan IBD-Nutrition Risk have since been designed specifically to screen for malnutrition in patients with IBD.^13^ The common goal of these MSTs is to risk stratify patients and identify those who could benefit most from interventions such as dietitian referrals, laboratory screening, initiation of nutrition supplements, or initiation of enteral or parenteral nutrition support.^12^

While improving malnutrition screening in hospitalized patients with IBD has been a focus of numerous quality improvement (QI) initiatives, there is currently a gap in the literature both in establishing the current state of malnutrition screening for patients with IBD in the outpatient setting and in identifying tangible ways in which malnutrition screening rates in the outpatient setting can be improved. Hwang et al. published a detailed algorithm to establish a malnutrition screening program for patients with IBD in the ambulatory setting and guide management for “at risk” patients (those with a modified MUST score ≥ 1).^12^ Gold et al. was one of the few groups to publish information about malnutrition screening rates for patients with IBD in the outpatient setting.^3^ However, these studies were conducted in the pre-COVID era, when the landscape of outpatient clinic visits was arguably much different than it is today, given that over the last several years increased adoption of telehealth, including virtual visits via telephone and video platforms,^14^, ^15^, ^16^, ^17^ has influenced how IBD care is delivered.

The purpose of this study is to assess the current state of outpatient malnutrition screening practices for IBD patients receiving care in gastroenterology (GI) clinics and evaluate risk factors for lack of malnutrition screening.

## Materials and Methods

### Study Design and Population

We performed a retrospective cohort study at the University of California Los Angeles (UCLA), a large academic health system that includes a mix of subspecialty clinics and community practices. Our study included adult patients with IBD already on advanced therapies who were seen in UCLA GI clinics between 2018 and 2024. The included patients’ most recent GI encounter with at least 6 months of follow-up data available after said encounter date was selected for chart review. All patients were confirmed to meet the inclusion criteria via manual chart review with institutional review board–approved protocols. Patients were excluded if they were less than 18 years old or if their current advanced therapy was started at an institution other than UCLA.

### Data Collection

The GI encounter date selected for data abstraction was the most recent outpatient GI encounter for each included patient with at least 6 months of subsequent follow-up data available after the selected encounter date. Patient demographic characteristics that were manually abstracted included age, sex, race, ethnicity, insurance type, body mass index (BMI), and smoking status. Clinical characteristics abstracted included IBD type, disease duration, disease behavior, disease location/extent, disease activity status at the time of the selected encounter date, whether they received care from an IBD specialist vs general gastroenterologist, and encounter type (telehealth vs in-person).

### Outcomes

We determined if malnutrition screening was performed at the patient’s most recent GI clinic encounter that had at least 6 months of existing follow-up time available from this encounter date. This was done through comprehensive chart review of notes from included patients’ GI encounters. We defined malnutrition screening as having been performed if there was a documented utilization of a standardized MST, physical examination pertaining to nutritional status, inquiry about weight loss, or inquiry about oral intake during the clinical encounter. We defined patients as being at risk of malnutrition if there was documentation of positive screening based on use of a MST, physical examination concerning for malnutrition, reported weight loss, or reported decrease in oral intake during the clinical encounter reviewed. If a patient was identified as being at risk of malnutrition, we determined whether nutrition labs and/or referral to a registered dietitian (RD) were ordered by the GI physician during the clinical encounter. Nutrition labs included obtaining one or more of the following tests: iron studies/ferritin, vitamin B12, folate, vitamin D, zinc, calcium, magnesium, albumin, vitamin E, vitamin A, and vitamin K. For patients at risk of malnutrition who were referred to a RD, we determined whether patients were seen by a RD within 6 months of the referral date.

### Root Cause Analysis

A root cause analysis (RCA) was performed to better understand why malnutrition screening may not have been performed by GI physicians during IBD-related clinic encounters. The RCA was created through utilization of both a Gemba walk, which included direct observation of employees in the clinic workplace and inquiry about typical workflows,^18^ and other interviews with relevant stakeholders. The term Gemba walk is derived from a Japanese term meaning “where the work occurs”.^18^ It is a learning practice in Lean methodology and the Six Sigma framework of QI that involves visiting a workplace to gain firsthand insight into an institution’s actual work processes and to better understand potential challenges and inefficiencies to inform QI interventions.^18^ This study’s Gemba walk was performed in both an IBD clinic as well as a general GI clinic and both in-person and telehealth encounters were observed. Stakeholder interviews were conducted with various GI attendings, GI fellows participating in IBD continuity clinic, and an RD in the Division of Digestive Diseases.

### Statistical Analysis

Categorical data were compared using chi-squared or Fisher's exact tests, where appropriate. Continuous variables were compared using the Student *t* test. Univariable and multivariable logistic regression were used to analyze whether malnutrition screening was performed and to evaluate potential risk factors for lack of malnutrition screening during clinical encounters. Multivariable models were adjusted for age, sex, race, IBD type, disease duration, active disease, extraintestinal manifestations, BMI, and visit type. Statistical significance was defined as an alpha threshold of 0.05. All statistical analyses were performed using Python 3.11 (Wilmington, Delaware).

## Results

There were 283 patients included in this retrospective study. Demographic and clinical characteristics of the included patients are summarized in Table 1. The mean age was 44.4 years (standard deviation [SD] 16.5), the mean BMI was 25.9 (SD 5.5), almost half of the patients (53.7%) were female, and the majority were White (62.9%) and non-Hispanic (75.5%). Almost all patients were insured with 17.3% having public insurance and 80.6% having private insurance. Half (50%) had Crohn’s disease (CD) and 46.8% had ulcerative colitis (UC). The mean disease duration was 13.4 years (SD 11.4) and 21.6% had recently active disease which was defined as having biochemical evidence of inflammation using local laboratory thresholds (calprotectin >50 μg/g stool or C-reactive protein >0.3 mg/dL) within 6 months of the GI clinic encounter date. There were 25.4% with extraintestinal manifestations of IBD. Most patients (70.7%) had their IBD-related GI clinic encounter via telehealth which was either a telephone visit or video visit.

**Table 1:** Patient Demographic and Clinical Characteristics

Variable	Overall cohort (N = 283)	Screened (N = 158)	Not screened (N = 125)	*P* Value
	N (%)	N (%)	N (%)	
Age, mean (SD)	44.4 (16.5)	44.1 (16.2)	44.9 (17.0)	1
Sex				
Male	131 (46.3)	68 (43.0)	63 (50.4)	.26
Female	152 (53.7)	90 (57.0)	62 (49.6)	
Race				
White	178 (62.9)	100 (63.3)	78 (62.4)	**<.01**
Black	11 (3.9)	3 (1.9)	8 (6.4)	
Asian	18 (6.4)	16 (10.1)	2 (1.6)	
Other	32 (11.3)	12 (7.6)	20 (16.0)	
Unknown	44 (15.5)	27 (17.1)	17 (13.6)	
Ethnicity				
Hispanic	36 (12.8)	17 (10.8)	19 (15.3)	.52
Non-Hispanic	213 (75.5)	122 (77.2)	91 (73.4)	
Unknown	33 (11.7)	19 (12.0)	14 (11.3)	
Insurance				
Public	49 (17.3)	25 (15.8)	24 (19.2)	.48
Private	228 (80.6)	128 (81.0)	100 (80.0)	
Uninsured	1 (0.4)	1 (0.6)	0 (0)	
Unknown	5 (1.8)	4 (2.5)	1 (0.8)	
BMI, mean (SD)	25.9 (5.5)	25.6 (5.5)	26.2 (5.5)	1
Smoking				
Current	11 (4.0)	9 (5.8)	2 (1.6)	.09
Former	67 (24.2)	32 (20.8)	35 (56.9)	
Never	199 (71.8)	113 (73.4)	86 (69.9)	
IBD type				
CD	140 (50.0)	90 (57.3)	50 (40.7)	**.01**
UC	131 (46.8)	61 (38.9)	70 (56.9)	
IC	9 (3.2)	6 (0.5)	3 (2.4)	
Disease duration, mean (SD)	13.4 (11.4)	12.8 (10.7)	14.2 (12.2)	1
CD behavior				
B1	50 (36.5)	34 (39.1)	16 (32.0)	.42
B2	36 (26.3)	19 (21.8)	17 (34.0)	
B3	15 (10.9)	9 (10.3)	6 (12.0)	
B2+B3	11 (8.0)	9 (10.3)	2 (4.0)	
Unknown	25 (18.2)	16 (18.4)	9 (18.0)	
CD location				
L1	38 (27.7)	24 (27.6)	14 (28.0)	.56
L2	31 (22.6)	20 (23.0)	11 (22.0)	
L3	54 (39.4)	36 (41.4)	18 (36.0)	
L4	6 (4.4)	2 (2.3)	4 (8.0)	
Unknown	11 (8.0)	8 (9.2)	3 (6.0)	
Perianal disease				
Yes	36 (25.9)	27 (30.3)	9 (18.0)	.16
No	103 (74.1)	62 (69.7)	41 (82.0)	
UC extent				
E1	7 (5.4)	2 (3.3)	5 (7.2)	.37
E2	37 (28.7)	15 (25.0)	22 (31.9)	
E3	85 (65.9)	43 (71.7)	42 (60.9)	
Extra-intestinal manifestations				
Yes	72 (25.4)	46 (29.1)	26 (20.8)	.14
No	211 (74.6)	112 (70.9)	99 (79.2)	
Recently active disease				
Yes	61 (21.6)	31 (19.6)	30 (24.0)	.46
No	222 (78.4)	127 (80.4)	95 (76.0)	
IBD specialist				
Yes	203 (71.7)	110 (69.6)	93 (74.4)	.46
No	80 (28.3)	48 (30.4)	32 (25.6)	
Encounter type				
In-person	83 (29.3)	57 (36.1)	26 (20.8)	**<.01**
Telehealth	200 (70.7)	101 (63.9)	99 (79.2)	

Bold values indicate significant variables with *P* value <.05.

Summary of demographic and clinical characteristics for the study’s overall cohort (n = 283), individuals who were screened for malnutrition (n = 158), and individuals who were not screened for malnutrition (n = 125).

**Figure 1:**
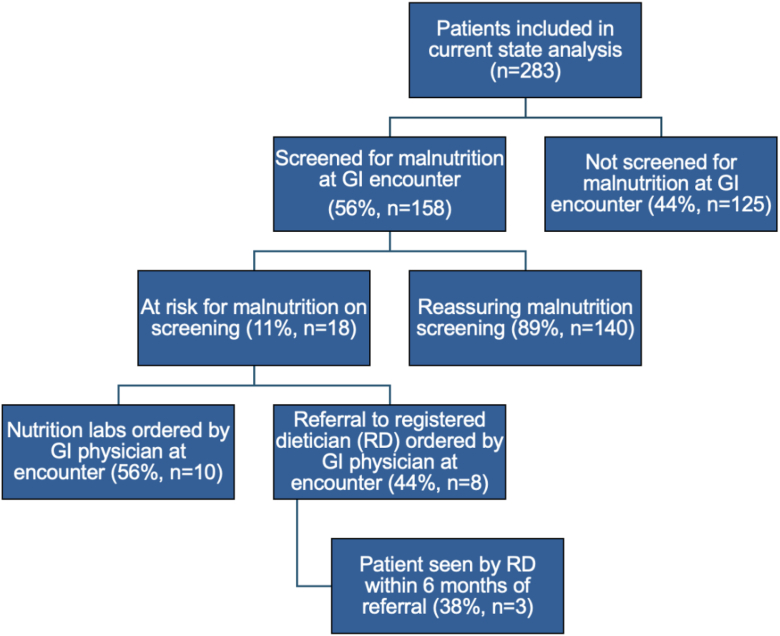
Current state analysis of ambulatory malnutrition screening for patients with IBD. Flow chart depicting the current state analysis of malnutrition screening for patients with IBD at outpatient IBD-related GI encounters (n = 283).

Figure 1 summarizes the outpatient malnutrition screening rate among GI physicians at ambulatory IBD-related encounters, rates of identified malnutrition risk, and subsequent screening result-directed interventions conducted. Malnutrition screening was performed at 56% of IBD-related GI encounters, either via a validated screening tool, inquiry about oral intake or about weight loss, physical examination, or some combination of the aforementioned. History of weight loss and/or poor oral intake on review of systems alone was the screening method used in a majority (66%) of encounters. A validated screening tool was used in 12% of these GI encounters. The only validated screening tool that was utilized was the MST. Physical examination was used to assess malnutrition risk in 7% of encounters. Screening rates at GI encounters remained stable in recent years, with screening occurring during 67% of reviewed encounters from 2021, 54% of reviewed encounters from 2022, and 58% of reviewed encounters from 2023.

Patient encounters that were reviewed were conducted by 2 IBD specialists and 19 general GI physicians. Malnutrition risk was identified in 11% of IBD-related encounters when GI physicians did screen for malnutrition. GI physicians ordered RD referrals 44% of the time and nutrition labs 56% of the time during encounters where malnutrition risk was identified via screening. Among the patients who had a RD referral ordered (n = 8), 38% saw a RD within 6 months of the referral order date.

Multivariable model results were used to determine potential risk factors for lack of malnutrition screening and are summarized in Table 2. Malnutrition screening was significantly less likely to be performed by GI physicians if the encounter was a telehealth visit (vs in-person visit, odds ratio [OR] 0.43, 95% confidence interval [CI] 0.23–0.80, *P* < .01). Malnutrition screening was more likely to be performed by GI physicians if the patient was Asian (vs White, OR 5.23, CI 1.13–24.33, *P* = .03). Malnutrition screening was less likely to be performed by GI physicians if the patient had UC or indeterminate colitis (IC). There was no significant difference in mean BMI between the racial groups. Age, sex, BMI, IBD type, extraintestinal manifestations, active disease, and receiving care from an IBD specialist (n = 2) vs general GI physician (n = 19) were not significantly associated with having malnutrition screening performed.

**Table 2:** Predictors of Having Malnutrition Screening Performed at Outpatient GI Encounters

Variables	Outcome: malnutrition screening performed
	Adjusted OR (95% CI)
Age, y	1.00 (0.98–1.01)
Sex	
Male	Reference
Female	1.45 (0.87–2.43)
Race	
White	Reference
Black	0.24 (0.06–1.01)
Asian	**5.23 (1.13 -24.33)**
Other	0.47 (0.21–1.06)
Unknown	1.43 (0.70–2.94)
IBD type	
CD	Reference
UC	**0.58 (0.35 – 0.98)**
Active disease	0.95 (0.51–1.75)
Extra-intestinal manifestations	1.58 (0.86–2.91)
BMI	0.99 (0.94–1.03)
Visit type	
In-person	Reference
Telehealth	**0.43 (0.23 - 0.80)**
IBD specialist	1.09 (0.59–2.03)

Bold values indicate significant variables with *P* value <.05.

Multivariable regression results for having malnutrition screening performed by GI, physicians at IBD-related ambulatory GI, encounters when adjusted for age, sex, race; IBD, type, active disease, presence of extraintestinal manifestations, BMI, visit type, and whether they were followed by an IBD, specialist vs general GI, physician.

CI, confidence interval; OR, odds ratio.

**Figure 2:**
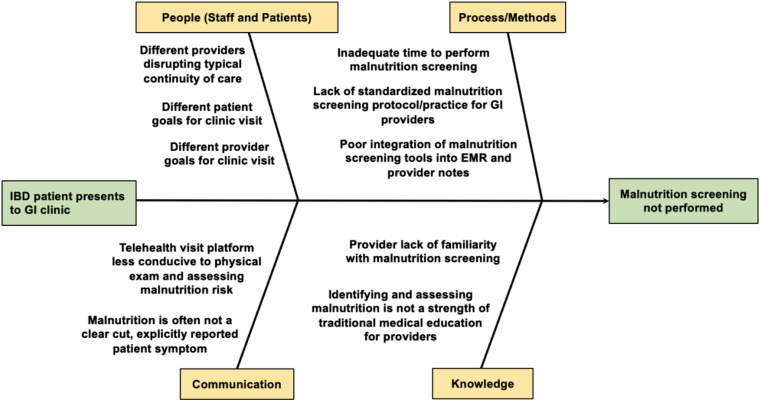
RCA for lack of malnutrition screening. RCA for lack of malnutrition screening performed by GI physicians at outpatient IBD-related GI encounters. RCA was created after Gemba walk and discussion with GI attendings, GI fellows participating in IBD continuity clinic, and a GI-registered dietitian. EMR, electronic medical record.

The second phase of this study was aimed at better understanding why malnutrition screening was not being performed at all IBD-related GI clinic encounters. RCA, which incorporated both observations from the Gemba walks and interviews with relevant stakeholders, identified several potential reasons for lack of malnutrition screening during IBD-related clinic encounters which are summarized in a fishbone diagram format in Figure 2. These reasons were organized into the following categories: people (both patients and clinic staff), process/methods, communication, and knowledge. Within the people category, differing goals for the clinic encounter between provider and patient was cited as a barrier for screening. GI fellows and attendings frequently cited barriers within the process/methods category as reasons for why malnutrition screening was not completed. Commonly physicians explained that they did not assess for malnutrition risk due to competing clinic demands with limited time to perform screening, lack of protocolization for malnutrition screening, and poor integration of MSTs into the electronic medical record. Within the communication category, they cited difficulty evaluating for and picking up on malnutrition risk given malnutrition is often not a clear cut, uniform, explicitly reported patient symptom. Additionally, GI physicians noted that telehealth visit platforms were less conducive to conducting a physical exam and gauging malnutrition risk. Within the knowledge category, GI physicians cited their lack of familiarity with malnutrition screening and assessing malnutrition risk as barriers for completing screening during GI encounters.

## Discussion

This study reveals the need for improvement in utilization of malnutrition screening among GI physicians for patients with IBD during ambulatory GI encounters. Patients were screened for malnutrition at slightly more than half (56%) of IBD-related GI encounters. When malnutrition screening was performed, an objective, validated MST was used only 12% of the time. Among those screened for malnutrition, GI physicians identified potential risk of malnutrition in nearly 1 of 9 (11%) IBD-related GI encounters. There was no significant difference in malnutrition screening practices between IBD specialists (n = 2) and general GI physicians (n = 19), however, this may be because our study was not powered sufficiently, especially with regards to IBD specialists, to see a difference between provider practices. Multivariable models revealed that patients with UC/IC were 42% less likely to be screened for malnutrition that patients with CD. One explanation for this may be that often patients with CD tend to have more symptoms and complications which may prompt GI physicians to ask about nutrition more often than they do for UC/IC patients. Another major finding of this study is that patients with telehealth encounters were 57% less likely to be screened for malnutrition than patients who had an in-person encounter.

Telehealth visits, which have become an increasingly more common modality for health-care delivery in the post-COVID-19 pandemic era,^17^ were strongly associated with lack of malnutrition screening. Our RCA suggested that one reason for this may be that telehealth visits are less conducive to assessing BMI and physical examination which often are used to help physicians gauge risk of malnutrition. However, prior literature has shown that having malnutrition and being overweight are not mutually exclusive but rather the two can coexist, and thus relying on vital signs to obtain a BMI measurement alone is not sufficient to accurately screen for malnutrition regardless of visit type.^19^ Although overweight patients can be malnourished, a low BMI measured during an in-person encounter is an overt clue to underlying malnutrition and lack of that clue during telehealth encounters may be a missed opportunity. Strategies to address this specific care gap are needed to ensure high quality care is maintained for the increasing number of ambulatory patients who are utilizing telehealth platforms, given 72% of GI encounters in our study were conducted via telehealth. While there are challenges to conducting a physical examination over telehealth platforms, the MST offers an alternative way to assess patients’ risk of malnutrition regardless of visit type. The MST is a quick and simple 2- or 3-question survey that involves asking about weight loss and appetite that has a sensitivity of 93% and specificity of 93% for malnutrition.^20^ Standardization of malnutrition screening for ambulatory IBD patients to include use of a validated screening tool, such as the MST, by the GI physician conducting the encounter would be helpful in eliminating the screening disparity across visit types.

It is well documented that malnutrition treatment leads to improved IBD outcomes; however, these treatments can only be initiated once malnutrition risk has been evaluated for and identified, which requires effective, widespread screening. The single prior study reporting a current state analysis of ambulatory malnutrition screening for IBD patients (Gold et al.) developed a QI intervention in which patients were screened for malnutrition at the beginning of clinic visits by medical assistants while assessing vital signs.^3^^,^^21^ While this proposed protocol was considered feasible and effective at the time, it does not address the lack of malnutrition screening in telehealth encounters, given that medical assistants do not typically interface with patients when visits are conducted via telehealth. Our study’s RCA suggests that implementing standardized malnutrition screening protocols for GI physicians to follow and integrating validated MSTs into the electronic medical record and provider notes may be beneficial, especially for use during telehealth encounters where physicians are often the only health-care team member interacting directly with patients. Moreover, addressing GI physician knowledge gaps in identifying and assessing malnutrition may also be helpful.

Once malnutrition risk has been evaluated for and identified, the next step is ensuring formal nutrition assessment by a RD and optimization of nutrition via dietary modifications, enteral support, or parenteral support. Our study revealed that even when patients were screened for malnutrition, found to be at risk of malnutrition by GI physicians, and had a RD referral appropriately placed by GI physicians in response to malnutrition screening, only 38% of the time were patients seen by a RD within 6 months of referral date. A significant barrier to patients receiving appropriate RD care is lack of insurance coverage. Although nutrition plays an integral role in digestive health, many insurance carriers do not cover RD visits and thus falls back on patients to shoulder this expense. Although our health system has multiple RDs solely focused on GI, there is an imbalance of supply and demand, which invariably leads to delay in care. This barrier is even further amplified in clinical practices that do not have access to RD care. Moreover, some patients may not perceive of the gravity of malnutrition risk or be ready to make significant changes in their dietary habits and thus may not pursue scheduling the RD visit.

Our study fills an existing gap in the current literature by establishing the current state of malnutrition screening practices for ambulatory IBD patients in the post-COVID-19 telehealth era. Gold et al. was first to demonstrate the feasibility and impact of a malnutrition screening program in ambulatory IBD patients by utilizing a modified MUST to streamline malnutrition screening.^3^ Prior to their QI initiative, they established the current state of malnutrition screening to be 3% based on 1 month of data.^3^ Strengths of our study are that it includes a much larger sample size over several years, providing a more robust and accurate estimate of the current state of the malnutrition screening rate in ambulatory IBD patients at a tertiary care center. Additionally, our study identifies a novel care gap for ambulatory IBD patients receiving care via telehealth. Moreover, to our knowledge, this is the first study to incorporate a RCA in the current state analysis of malnutrition screening in the ambulatory IBD patient population which will be useful in guiding development and implementation of effective, sustainable QI interventions to improve malnutrition screening rates.

Despite the aforementioned strengths of this study, there are also some potential limitations. Firstly, our study captures data from only a single clinic encounter per IBD patient, thus it is possible that although malnutrition screening was not performed at the selected clinic encounter, it may have been performed at prior encounters. However, malnutrition is often a recurrent, insidious diagnosis and ideally, IBD patients who are inherently higher risk for malnutrition should be continuously re-evaluated for malnutrition at each GI clinic encounter to ensure this crucial diagnosis is not missed. Secondly, given the retrospective design of our study, there is the chance for unaccounted confounders. Thirdly, the study was conducted at a single academic, tertiary care center with dedicated IBD clinics and specialists as well as RDs, which is not necessarily reflective of other practice settings that care for patients with IBD. It is likely that a general GI physician in a community clinic setting will not have the resources to refer patients to RDs and may be less likely to screen for malnutrition given there are fewer available screening-directed interventions. Overall, these factors may limit the generalizability of our results. Lastly, while our sample size was larger than that in prior studies, it was not racially or ethnically diverse. While we found that Asian patients were much more likely to be screened for malnutrition, the sample size of Asian patients was too small to be able to draw any meaningful conclusions. We evaluated for differences in BMI and other demographic and clinical factors between race groups but did not identify an explanation for the higher rate of malnutrition screening among Asian patients.

## Conclusion

Malnutrition screening rates for ambulatory patients with IBD can and should be improved, especially for the large number of patients relying on telehealth modalities to receive their outpatient IBD care. The next phase of our QI efforts will include the design and implementation of the following QI interventions based on the findings of our current state analysis and RCA: (a) patient previsit questionnaire with integrated MST, (b) standardized physician IBD note templates that integrate the MST and its results, and (c) physician education and communication regarding importance of malnutrition screening at all IBD-related ambulatory GI encounters. We will then evaluate the impact of these interventions on screening rates and related clinical outcomes to complete the first Plan-Do-Study-Act cycle of our study. Given the importance of adequate nutrition for maintaining physiologic function, we anticipate that improvement in malnutrition screening would lead to improved clinical outcomes among patients with IBD.
